# Investigation of Helium Behavior in RAFM Steel by Positron Annihilation Doppler Broadening and Thermal Desorption Spectroscopy

**DOI:** 10.3390/ma11091523

**Published:** 2018-08-24

**Authors:** Zhenyu Shen, Liping Guo, Weiping Zhang, Shuoxue Jin, Xingzhong Cao, Yunxiang Long, Yaxia Wei

**Affiliations:** 1 Hubei Nuclear Solid Physics Key Laboratory, Key Laboratory of Artificial Micro- and Nano-structures of Ministry of Education and School of Physics and Technology, Wuhan University, Wuhan 430072, China; shenzy@whu.edu.cn (Z.S.); zhangweiping@whu.edu.cn (W.Z.); kobe-long@whu.edu.cn (Y.L.); weiyx@whu.edu.cn (Y.W.); 2 Key Laboratory of Nuclear Radiation and Nuclear Energy Technology, Institute of High Energy Physics, Chinese Academy of Sciences, Beijing 100049, China; jinshuoxue@ihep.ac.cn (S.J.); caoxzh@ihep.ac.cn (X.C.)

**Keywords:** reduced-activation ferritic/martensitic steel, helium, positron annihilation spectroscopy, thermal desorption spectroscopy

## Abstract

The behavior of helium in reduced-activation ferritic/martensitic steels was investigated systematically with positron annihilation Doppler broadening measurement and thermal desorption spectroscopy. Specimens were irradiated with helium ions with different energies to various fluences at different temperatures. A threshold fluence was observed above which the rate of formation and growth of helium bubbles dramatically increased. Irradiation at higher temperature could suppress the formation and growth of He_n_V_m_ clusters with low binding energies and enhance that of helium bubbles and He_n_V_m_ clusters with high binding energies. Different changes of S parameters were observed in various depth after the irradiation temperature was increased from 523 K to 723 K. Irradiation of 18 keV-He^+^ enhanced the growth of He_n_V_m_ clusters and helium bubbles compared with 100 keV-He^+^ irradiation. A possible mechanism is discussed.

## 1. Introduction

Reduced-activation ferritic/martensitic (RAFM) steels are considered as one of the most promising candidates as the structural materials for future fusion reactors for their excellent swelling resistance and thermal-physical and thermomechanical properties [1]. Under fusion conditions, the structural materials are confronted with the irradiation of neutrons with high energy. Meanwhile, due to (n, α) and (n, p) nuclear transmutation reactions, considerable helium and hydrogen are introduced into the structural materials, which are involved in the evolution of microstructure and affect the mechanical properties of materials [2,3]. In particular, helium atoms are reported to enhance the shift of ductile-to-brittle transition temperature (DBTT), swelling, and irradiation hardening in steels [4,5].

It is widely acknowledged that helium atoms can be trapped by vacancies, leading to the formation of He–V clusters. He–V clusters keep absorbing helium atoms and vacancies, finally resulting in the nucleation of helium bubbles, which are blamed for the material degradation [6]. For this reason, a thorough understanding of the behaviors of He and He–V clusters in RAFM steels at different irradiation conditions is important. Many calculation studies based on first-principle calculation [7,8,9], modular dynamics [10], and kinetic Monte-Carlo [11,12] reported results about the stability and mobility of He–V clusters in α-Fe. Some experiments using transmission electron microscopy (TEM) studied helium bubbles’ behavior in RAFM steels [13,14,15]. However, owing to the limit of the resolution power of TEM on helium, observation of small defects and defect clusters containing both helium atoms and vacancies is beyond its ability.

Positron annihilation Doppler broadening (DB) measurement is a powerful technique to characterize vacancy-type defects in materials. There have been some works [16,17,18,19,20] focused on the behavior of helium in RAFM steels based on positron annihilation DB measurement. Qiu et al. [17,18] studied the effects of He irradiation to various fluences at room temperature on RAFM steels. Some studies [16,19,20] focused on helium behavior in RAFM steels at different temperatures. However, the reported results are not yet self-consistent and the mechanism remains unclear, especially for the dependence on temperature. 

In present work, helium ion irradiations with different energies to various fluences and at different temperatures have been conducted on RAFM steels. Positron annihilation DB measurement together with thermal desorption spectroscopy (TDS) was employed to investigate the behavior of helium in RAFM steels.

## 2. Materials and Methods 

**Figure 1:**
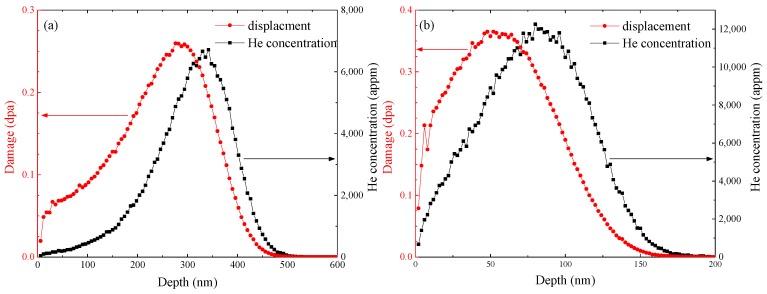
Depth profile of helium concentration and damage events in RAFM steels after irradiation to 1 × 10^20^ m^−2^ with (**a**) 100 keV-He^+^ and (**b**) 18 keV-He^+^.

The RAFM steel used in the present study has been reported in [21] and the chemical composition of the steel is presented in Table 1. Its production procedure was described in detail in our previous study [22]. It was observed that dislocations existed in martensitic lath, and carbides or carbonitrides formed along lath boundaries and grain boundaries [21]. The average lath width of this steel is 0.64 µm [21]. After being cut into square sheets of 10 × 10 × 0.5 mm, all the samples were polished mechanically with silicon carbide paper with the grades of 600–2000 before ion irradiation. The helium ion irradiation experiments were conducted with the ion implanter at the Accelerator Laboratory of Wuhan University [23]. The irradiation conditions are described in detail in Table 2. Based on calculation with SRIM-2013 software [24] using a displacement energy of 40 eV [25], the depth profiles of damage and helium concentration introduced by helium ion irradiation with energies of 18 keV and 100 keV to the fluence of 1 × 10^20^ m^−2^ are shown in Figure 1, respectively.

Positron annihilation DB measurements were conducted at the slow positron beam facility in the Institute of High Energy Physics. The positron beam energy ranged from 0.18 to 20 keV. The average implanted depths of the slow positrons were calculated by the empirical equation [26,27]:

(1) $$Z\left(E\right)=\left(\frac{4\times 10^{4}}{\rho }\right)E^{1.6}$$

where *Z*(*E*) is the depth below surface in units of nm, *E* is the incident energy of the slow positron in keV, and *ρ* is the density of RAFM steel in kg/m^3^. The S and W parameters are defined as the ratios of the counts in the central low momentum area (510.2–511.8 keV) and the two flanking high momentum regions (514.83–518.66 keV and 503.34–507.17 keV) to the total counts in the DB spectra, respectively. TDS analysis experiments were also conducted in the Institute of High Energy Physics and performed from room temperature to 1523 K using infrared radiation heating. A quadrupole mass spectrometer and a thermocouple were used to detect released He atoms and measure the temperature of the specimen, respectively. The heating rate was fixed at 1 K/s, which was controlled by a PID (proportion, integral, derivative) feedback loop. The minimum detection limit of helium desorption rate was 2.45 × 10^9^ s^−1^. During the experiments, a high vacuum was maintained at 10^−6^ Pa.

## 3. Results and Discussion

### 3.1. RAFM Steels Irradiated to Different Fluences

**Figure 2:**
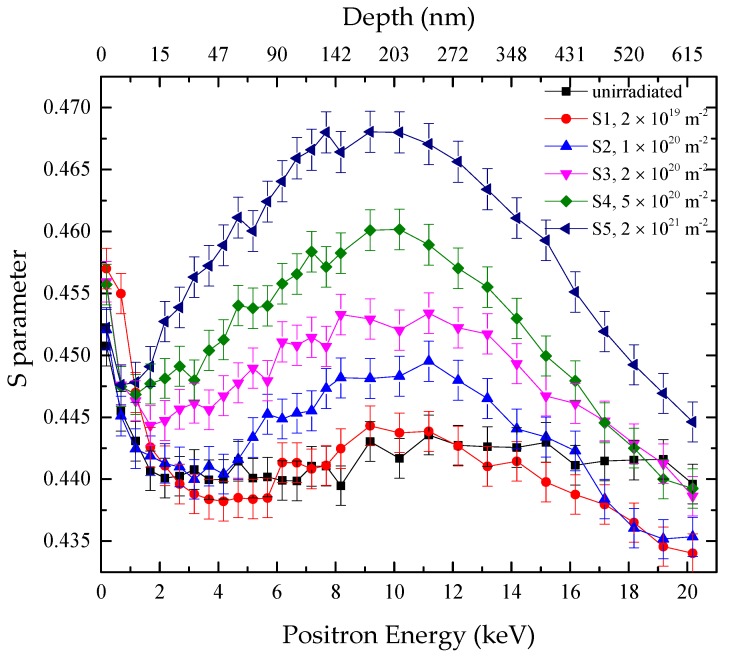
S–E curves for RAFM steels without irradiation and irradiated at 523 K to different fluences (2 × 10^19^, 1 × 10^20^, 2 × 10^20^, 5 × 10^20^, and 2 × 10^21^ m^−2^).

Figure 2 shows the S parameters of the specimens before irradiation and after irradiation with 100 keV-He^+^ at 523 K to different fluences (specimens S1–S5). The average implanted depths of positrons according to Equation (1) are shown on the top x-axis in Figure 2. At the surface, the S parameters of all the specimens decreased with the increase of energy of incident positron. This phenomenon resulted from the diffusion of positrons to the surface and formation of ortho-positronium [28].

For the samples irradiated with He^+^, the S–E curves all followed a similar trend. At the surface, the S parameters decreased with the increase of positron energy, as mentioned earlier. In the bulk region, with the increase of positron energy, the S values increased initially and then decreased, forming a peak standing in the depth of about 170–230 nm. It was also observed that the higher the fluence, the larger were the S parameters. The results are expected and can be explained by the introduction of vacancies and vacancy clusters by irradiation of helium ions. The formation of vacancy-type defects increased the S parameters of the specimens. With higher irradiation fluence, more vacancy-type defects were introduced into the specimens, leading to larger values of the S parameters. The peak of the displacement depth profile introduced by 100 keV-He^+^ was at about 270 nm according to the calculation of SRIM-2013, as shown in Figure 1, not coincident with the peaks of the S–E curves. The discrepancy can be owing to the formation of helium–vacancy clusters. When positrons were captured by helium–vacancy clusters, the values of the S parameters were smaller compared to those for vacancy-type defects. As shown in Figure 1, the peak position of helium concentration was deeper from the surface compared with that of displacement. It may result in the noncoincidence between peaks of the S–E curves and the depth profile of displacement. For most of the specimens after irradiation, the S parameters were higher than those of the unirradiated specimen. However, for the sample S1 irradiated to a low fluence (2 × 10^19^ m^−2^), the situation was inversed in regions at depths from 25 nm to 82 nm and above 270 nm. It may result from the capture of intrinsic vacancies by implanted helium atoms.

**Figure 3:**
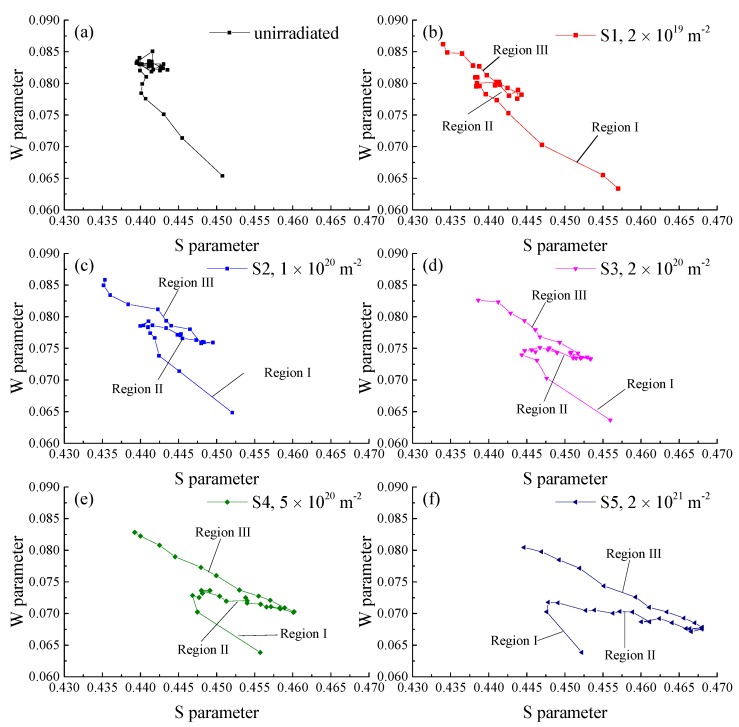
S–W curves for (**a**) the unirradiated specimen and specimens (S1–S5) irradiated at 523 K to different fluences: (**b**) 2 × 10^19^, (**c**) 1 × 10^20^, (**d**) 2 × 10^20^, (**e**) 5 × 10^20^, and (**f**) 2 × 10^21^ m^−2^.

Figure 3 shows the S–W plots of specimens without irradiation and irradiated with 100 keV-He^+^ at 523 K to various fluences (specimens S1–S5). Information of the mechanism of positron annihilation can be revealed by the slopes of the S–W plots, which can be used to identify the types of defects that are trapped by positrons in materials [18]. From Figure 3, it was observed that the annihilation mechanisms of irradiated samples were all similar. The S–W plots could be divided into three regions, ordered as Region I, II, and III from surface to bulk. Different regions are marked in Figure 3. At the surface and in the deepest region, the S–W plots presented similar slopes, which were nearly parallel with that of the unirradiated specimen. The slope may reveal the existence of vacancies and vacancy clusters. In the middle region, the slopes of plots were not observed in the S–W plot of the unirradiated specimen. It suggested that a type of defects newly formed after irradiation, which was coincident with the discussion above, i.e., after irradiation, helium–vacancy clusters formed in the material. A cluster of points was observed in the S–W plot of the unirradiated specimen. It may originate from the annihilation of positrons with electrons of matrix atoms.

**Figure 4:**
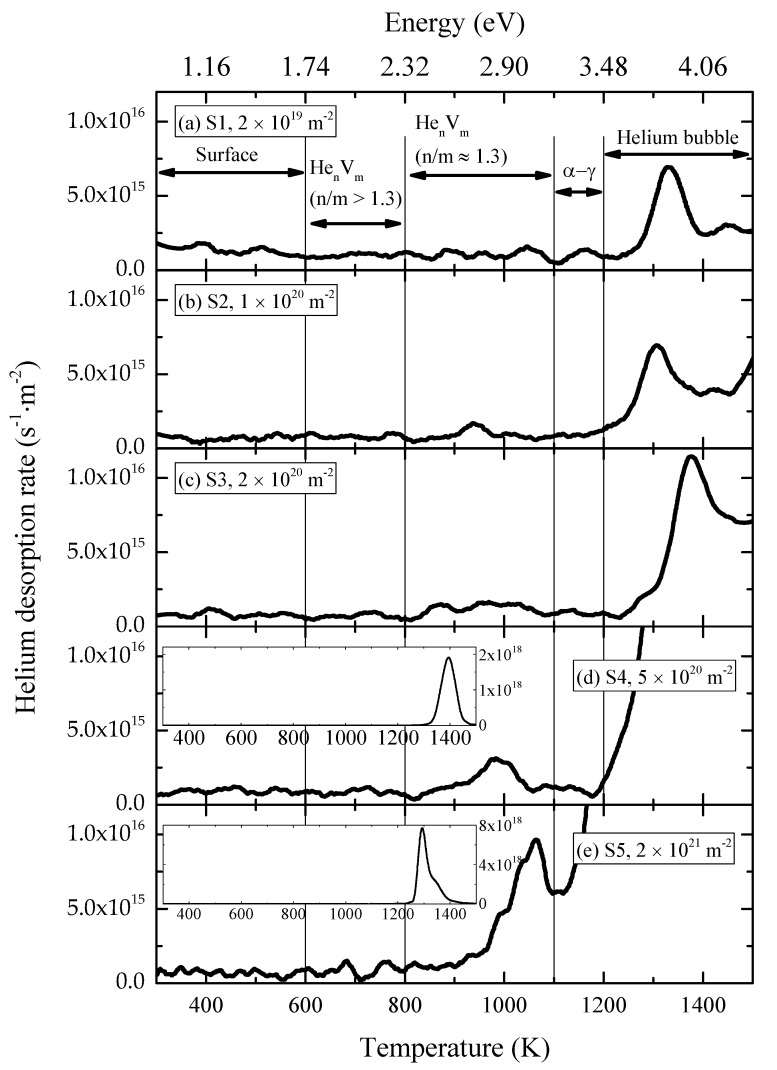
Desorption spectra in specimens irradiated with 100 keV-He^+^ at 523 K to various fluences: (**a**) 2 × 10^19^, (**b**) 1 × 10^20^, (**c**) 2 × 10^20^, (**d**) 5 × 10^20^, and (**e**) 2 × 10^21^ m^−2^.

Figure 4 shows the helium thermal desorption spectra for the specimens irradiated with 100 keV-He^+^ to different fluences at 523 K (specimens S1–S5). Based on the first-order dissociation model, the desorption rate of helium can be expressed as follows [10,29]:

(2) $$\frac{dN}{dt}=-k_{0}Nexp\left(-\frac{E}{k_{B}T}\right)$$

where *N* is the amount of helium atoms that remain in the specimen, $k_{0}$ is the jumping frequency (on the order of 10^13^ s^−1^), *E* is the dissociation energy of helium from the trap, and *k_B_* is the Boltzmann constant. A relationship between the helium desorption peak temperature *T* and the dissociation energy of helium from the trap *E* can be calculated according to Equation (2), as follows [10]:

(3) E=0.0029T

The dissociation energy of helium calculated according to Equation (3) is shown in the top axis of Figure 4.

The dissociation energy of helium from He_n_V_m_ clusters generally decreases, while that of vacancy increases with the increase of the ratio of n/m. It is suggested in the literature [10] that the configuration of He_n_V_m_ clusters with highest stability could be provided by the crossover of curves of dissociation energies of helium atoms and vacancies from He_n_V_m_ clusters depending on the ratio of n/m. According to calculation results [7,30], the most stable He_n_V_m_ cluster corresponds to n/m of about 1.3 in α-Fe. It is also shown in the literature [7,30] that for He_n_V_m_ clusters with n/m ≈ 1.3, the dissociation energy of helium ranges from 2.3 to 3 eV and the lowest dissociation energy of helium from He_n_V_m_ clusters is about 1.8 eV in α-Fe. Based on these facts, the helium thermal spectra could be divided into five regions. Region I ranges from 300 K to 600 K, region II ranges from 600 K to 800 K, region III ranges from 800 K to 1100 K, region IV ranges from 1100 K to 1200 K, and region V ranges from 1200 K to 1500 K. According to Equation (3), the dissociation energies of helium from the trap for each region can be estimated as follows: 0.87 eV to 1.74 eV for region I, 1.74 eV to 2.32 eV for region II, 2.32 eV to 3.19 eV for region III, 3.19 eV to 3.48 eV for region IV, and 3.48 eV to 4.35 eV for region V, respectively. Each region can be characterized as follows:

1. Region I: helium dissociated from surface;
2. Region II: helium dissociated from He_n_V_m_ clusters with n/m larger than 1.3;
3. Region III: desorption of stable He_v_V_m_ clusters with n/m of about 1.3;
4. Region IV: α–γ transformation corresponding desorption;
5. Region V: migration of helium bubbles.

**Figure 5:**
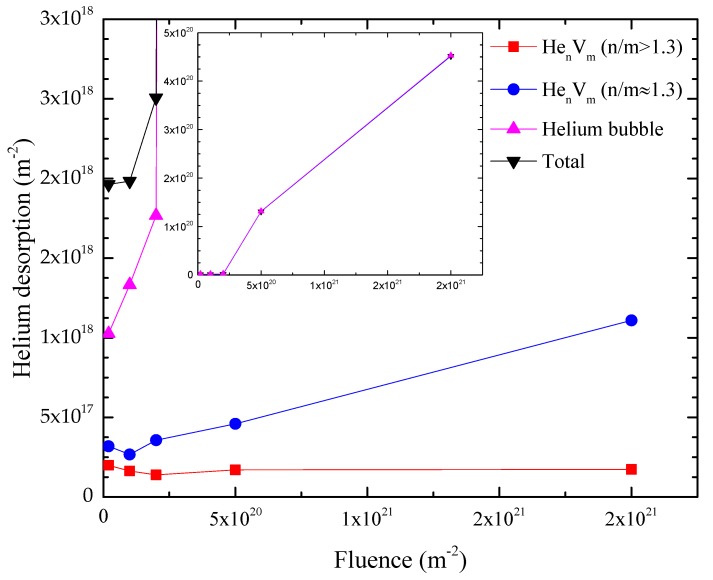
Amounts of helium released from various defect clusters and total helium amount released in specimens irradiated at 523 K to various fluences.

At present, desorption caused by α–γ transformation is considered as owing to structural instability around helium bubbles during transformation [31]. For this reason, the sum of the amounts of helium absorbed in regions IV and V corresponds to helium bubbles. Figure 5 shows the amounts of helium desorbed from different defects and total released helium after irradiation with 100 keV-He^+^ to different fluences at 523 K. The concentrations of He_n_V_m_ clusters and helium bubbles are closely related with these amounts of desorbed helium. The illustration in the figure is with a larger scale of the y-axis. With increase of the fluence, the amount of helium dissociated from He_n_V_m_ (n/m > 1.3) clusters is nearly kept constant, while those from He_n_V_m_ (n/m ≈ 1.3) clusters and helium bubbles increased. In other words, compared with He_n_V_m_ (n/m ≈ 1.3) clusters and helium bubbles, the formation and growth of He_n_V_m_ (n/m > 1.3) clusters saturated at a lower fluence. It was also observed that the increase in the rate of the amount of helium released from helium bubbles shifted dramatically when the fluence reached 2 × 10^20^ m^−2^.

The above results could be explained qualitatively with a rate equation. The changing rate of concentration of He_n_V_m_ clusters could be described by an equation as follows:

(4) $$\begin{array}{l} \frac{dC_{He_{n}V_{m}}}{dt}\\ =\left(k_{He_{n-1}V_{m}+He}^{+}C_{He}C_{He_{n-1}V_{m}}-k_{He_{n}V_{m}+He}^{+}C_{He}C_{He_{n}V_{m}}\right)\\ -\left(k_{H_{n}V_{m}-He}^{-}C_{He_{n}V_{m}}-k_{H_{n+1}V_{m}-He}^{-}C_{He_{n+1}V_{m}}\right)\\ +\left(k_{He_{n}V_{m-1}+V}^{+}C_{V}C_{He_{n}V_{m-1}}-k_{He_{n}V_{m}+V}^{+}C_{V}C_{He_{n}V_{m}}\right)\\ -\left(k_{H_{n}V_{m}-V}^{-}C_{He_{n}V_{m}}-k_{H_{n}V_{m+1}-V}^{-}C_{He_{n}V_{m+1}}\right)\\ -\left(k_{He_{n}V_{m}+I}^{+}C_{I}C_{He_{n}V_{m}}-k_{He_{n}V_{m+1}+I}^{+}C_{I}C_{He_{n}V_{m+1}}\right)\\ +\left(k_{H_{n}V_{m-1}-I}^{-}C_{He_{n}V_{m-1}}-k_{H_{n}V_{m}-I}^{-}C_{He_{n}V_{m}}\right)\\ -\underset{S}{\sum }K_{S}C_{He_{n}V_{m}}D_{He_{n}V_{m}}, \end{array}$$

where $C_{X}$ is the concentration of the defect or defect cluster *X*, $k_{He_{n}V_{m}+Y}^{+}$ represents the absorption rate coefficient between He_n_V_m_ clusters and defects *Y*, $k_{He_{n}V_{m}-Y}^{-}$ is the emission rate coefficient of defect *Y* from He_n_V_m_ clusters, $K_{S}$ is the sink strength of sink *S*, and $D_{He_{n}V_{m}}$ is the diffusion coefficient of the He_n_V_m_ cluster. *Y* can be helium atoms, vacancies, or self-interstitial atoms (SIAs). *S* can be precipitate interfaces, grain boundaries, or dislocations. Owing to low diffusion coefficients of He_n_V_m_ clusters, the last term could usually be neglected. $k_{He_{n}V_{m}+Y}^{+}$ and $k_{He_{n}V_{m}-Y}^{-}$ can be expressed as follows, respectively [32]:

(5) $$k_{He_{n}V_{m}+Y}^{+}=4\pi r_{He_{n}V_{m}}D_{Y}$$

(6) $$k_{He_{n}V_{m}-Y}^{-}=\frac{4\pi r_{He_{n}V_{m}-Y}D_{Y}exp\left(-\frac{E_{He_{n}V_{m}-Y}^{b}}{k_{B}T}\right)}{V_{at}}$$

where $r_{He_{n}V_{m}}$ is the cluster radius, $E_{He_{n}V_{m}-Y}^{b}$ is the binding energy between the He_n_V_m_ cluster and defect *Y*, and *V_at_* is the atomic volume. $D_{Y}$ can be expressed as follows [32]:

(7) $$D_{Y}=D_{0}\exp\left(-\frac{E_{Y}^{m}}{k_{B}T}\right)$$

where $D_{0}$ is the prepotential factor and $E_{Y}^{m}$ is the migration energy of defect *Y*. Equation (7) also applies to $D_{He_{n}V_{m}}$.

The calculation results [7,30] show that the binding energy of a helium atom with He_n_V_m_ (n/m > 1.3) clusters is smaller than that with He_n_V_m_ (n/m ≈ 1.3) clusters, which means that the emission coefficient of helium from He_n_V_m_ (n/m > 1.3) clusters is larger than that from He_n_V_m_ (n/m ≈ 1.3) clusters according to Equation (6). It could explain the observation that the formation and growth of He_n_V_m_ (n/m > 1.3) clusters saturated at a lower fluence compared with He_n_V_m_ (n/m ≈ 1.3) clusters and helium bubbles. According to Equation (4), the growth of He_n_V_m_ clusters also depends on the concentration of relatively smaller clusters, $C_{He_{n-1}V_{m}}$ and $C_{He_{n}V_{m-1}}$; i.e., the formation of helium bubbles depends on the concentration of He_n_V_m_ clusters. When the fluence was low, the concentration of He_n_V_m_ clusters was small, limiting the formation of helium bubbles. As the irradiation fluence increased, the concentration of He_n_V_m_ clusters also increased and the limitation on the formation of helium bubbles no longer existed. On the other hand, according to Equation (5), the absorption rate of helium atoms and vacancies by helium bubbles is higher than that of He_n_V_m_ clusters, since the radius of helium bubbles is larger compared with that of He_n_V_m_ clusters. The emission rates of helium atoms and vacancies from helium bubbles is smaller than that those from He_n_V_m_ clusters because the binding energy of a helium atom and vacancy with helium bubbles is larger than that with He_n_V_m_ clusters with the same n/m [7,33]. Based on these facts, when fluence was high enough and limitation on the formation of helium bubbles owing to the concentration of He_n_V_m_ clusters no longer existed, the formation and growth of helium bubbles played the dominant role. It resulted in the observation of the shift of increase in rate of the amount of helium released from helium bubbles when the fluence reached 2 × 10^20^ m^−2^, as shown in Figure 5.

### 3.2. RAFM Steels Irradiated at Different Temperatures

**Figure 6:**
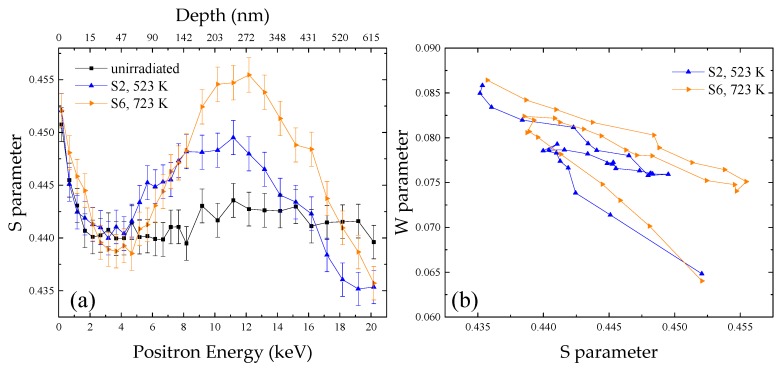
(**a**) S–E curves and (**b**) S–W curves for RAFM steels irradiated to the fluence of 1 × 10^20^ m^−2^ at different temperatures (523 and 723 K).

Figure 6a shows the S–E curves of the unirradiated specimen and the specimens irradiated to the same fluence (1 × 10^20^ m^−2^) at 523 K (specimen S2) and 723 K (specimen S6). It was observed in the figure that the S parameters for S6 were smaller from 15 to 133 nm and higher in the rest of the regions compared with those of S2.

**Figure 7:**
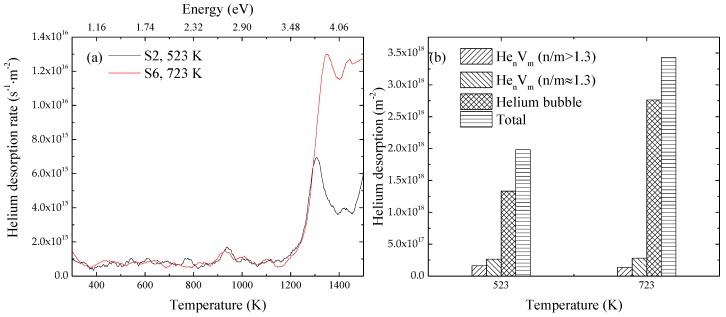
(**a**) Desorption spectra in specimens irradiated with 100 keV-He^+^ at 523 K and 723 K to 1 × 10^20^ m^−2^; (**b**) amounts of helium released from various defect clusters and total helium amount released in specimens irradiated with 100 keV-He^+^ at 523 K and 723 K to 1 × 10^20^ m^−2^.

The helium thermal desorption spectra for the specimen S2 and S6 are shown in Figure 7a, and the amounts of helium absorbed from various defects are shown in Figure 7b. The amount of helium released from He_n_V_m_ (n/m > 1.3) clusters in S6 was slightly smaller, while that of formed helium bubbles was significantly larger compared with S2. The amount of helium dissociated from He_n_V_m_ (n/m ≈ 1.3) clusters in S6 was also slightly larger. It meant that the formation and growth of He_n_V_m_ (n/m > 1.3) clusters was suppressed, while those of He_n_V_m_ (n/m ≈ 1.3) clusters and helium bubbles were both enhanced when the irradiation temperature changed from 523 K to 723 K. In our previous study, RAFM steels were irradiated with He^+^ to 1.6 × 10^20^ m^−2^ at 523 K and 723 K and examined with TEM [15]. Bubbles were observed in the specimen irradiated at 723 K; in contrast, no bubbles were observed in the specimen irradiated at 523 K. This is coincident with the results in the present study.

The emission rate of helium from He_n_V_m_ (n/m > 1.3) clusters is larger than that from He_n_V_m_ (n/m ≈ 1.3) clusters, as discussed above. On the other hand, a higher temperature also contributes to a higher emission rate, according to Equation (6). When the irradiation temperature changed from 523 K to 723 K, the combined higher temperature and lower binding energy suppressed the formation and growth of He_n_V_m_ (n/m > 1.3) clusters. Because the formation and growth of He_n_V_m_ (n/m > 1.3) clusters was suppressed, helium atoms and vacancies making up these clusters were left in the lattice under irradiation, leading to the increase of the concentrations of helium atoms and vacancies. This increase contributed to higher absorption rates of helium atoms and vacancies with He_n_V_m_ (n/m ≈ 1.3) and helium bubbles. The higher temperature also resulted in the increase of *D_He_* and *D_V_*, which meant larger absorption coefficients based on Equation (5). These facts suggested that the growths of He_n_V_m_ (n/m ≈ 1.3) clusters and helium bubbles were enhanced when the temperature changed from 523 K to 723 K. It resulted in the decrease of the amount of helium released from He_n_V_m_ (n/m > 1.3) and increase of that from He_n_V_m_ clusters (n/m ≈ 1.3) and helium bubbles, as observed in Figure 7b. Because He_n_V_m_ clusters with n/m ≈ 1.3 are the most stable, the average n/m of formed He_n_V_m_ clusters became closer to 1.3 after the increase of the irradiation temperature. 

According to SRIM-2013 calculation, after 100 keV-He^+^ irradiation, the ratio of concentrations of vacancies to helium atoms decreases with the increase of depth. In the region at depth above 133 nm, where the ratio between concentrations of helium atoms and vacancies introduced by irradiation was relatively higher, it was quite possible that the average ratio of n/m of formed He_n_V_m_ clusters was larger than 1.3 after irradiation at 523 K. After irradiation at 723 K, the average ratio of n/m of formed He_n_V_m_ clusters became smaller, which led to the increase of the S parameter, leaving some helium atoms in the lattice. In contrast, for the depth at 15 to 133 nm, the ratio between concentrations of helium atoms and vacancies introduced by irradiation was lower, suggesting that the average n/m of formed He_n_V_m_ clusters at 523 K was smaller than 1.3. When the irradiation temperature increased to 723 K, the emission of vacancies from He_n_V_m_ (n/m < 1.3) clusters was enhanced and the formation of He_n_V_m_ (n/m < 1.3) clusters was suppressed, hence the average n/m of formed He_n_V_m_ clusters became larger and closer to 1.3, leaving some vacancies in the lattice. Since the temperature was higher, the diffusivities of helium atoms were enhanced. In the depth above 133 nm, the helium atoms left in lattice diffused to the shallow region and captured the vacancies there. The combined increase of the average n/m of the He_n_V_m_ clusters and the trap of helium atoms by vacancies contributed to the decrease of the S parameters in these depths.

Figure 6b shows the S–W plots of the samples S2 and S6. Three regions could still be observed in the S–W plot for the samples S6, irradiated at 723 K. However, compared with specimen S2, a slight difference of the slopes of the surface and middle regions could be observed in S6. It originated from the difference of the average n/m of He_n_V_m_ clusters formed after irradiation at different temperatures, as discussed above.

### 3.3. RAFM Steels Irradiated with Helium Ions with Different Energies

**Figure 8:**
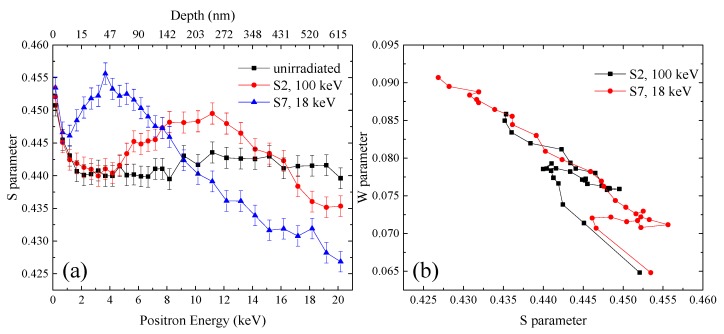
(**a**) S–E curves and (**b**) S–W curves for RAFM steels irradiated to the fluence of 1 × 10^20^ m^−2^ with 18 keV-He^+^ and 100 keV-He^+^.

Figure 8a shows the S–E curves of the samples irradiated to 1 × 10^20^ m^−2^ at 523 K with 18 keV-He^+^ (specimen S7) and 100 keV-He^+^ (specimen S2), respectively. The S–E curve of the unirradiated specimen is also shown. Compared with the specimen S2, the position of the peak of the S–E curve of the specimen S7 stood at the depth of about 40 nm, which was closer to the surface, and the height of peak was also higher. It was coincident with the calculation result of SRIM-2013 as shown in Figure 1: the peak of displacements produced by 18 keV-He^+^ was located at the depth of about 66 nm, much closer to the surface than that produced by 100 keV-He^+^, and peak damage dose produced by 18 keV-He^+^ was also higher than that produced by 100 keV-He^+^, meaning larger values of the peak. From Figure 8a, it was observed that the values of the S parameters at the depth above 177 nm in the specimen S7 were smaller than that in the unirradiated specimen. Actually, at 523 K, a proportion of the implanted helium atoms diffused into the nonimplanted bulk and trapped intrinsic vacancies, leading to the decrease of the concentration of vacancies and hence decrease of the S parameters.

Figure 8b provides the S–W plots of the specimens S2 and S7. The S–W plot of the specimen S7 could still be divided into three regions. However, the middle region was closer to surface compared with the specimen S2. Because the peaks of displacements and helium atoms introduced by 18 keV-He^+^ were closer to surface, it was reasonable that He_n_V_m_ clusters formed during irradiation were distributed closer to the surface.

**Figure 9:**
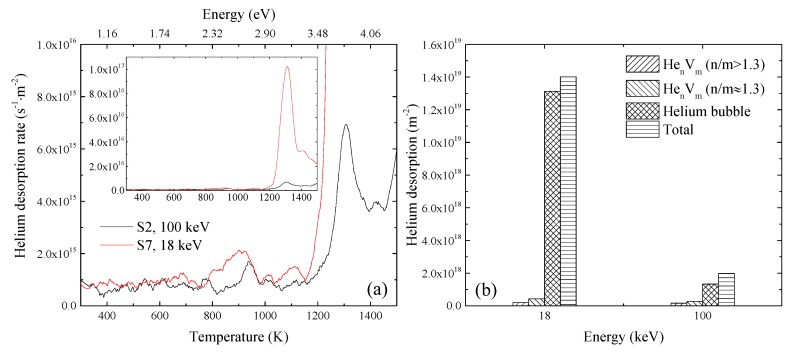
(**a**) Desorption spectra in specimens irradiated with 100 keV-He^+^ and 18 keV-He^+^ at 523 K to 1 × 10^20^ m^−2^; (**b**) amounts of helium released from various defect clusters, and total helium amount released in specimens irradiated with 100 keV-He^+^ and 18 keV-He^+^ at 523 K to 1 × 10^20^ m^−2^.

The thermal desorption spectra of helium for the specimens S2 and S7 are shown in Figure 9a, and the amounts of helium absorbed from various defects in the two specimens are shown in Figure 9b. It was observed that the amounts of helium desorbed from each type of defect clusters in the specimen S7 was higher than those in the specimen S2. As mentioned earlier, 18 keV-He^+^ irradiation introduced higher damage dose and helium concentration, meaning larger *C_He_* and *C_V_*, which was helpful for the formation and growth of He_n_V_m_ clusters and helium bubbles. However, the amounts of helium desorbed from sample S7 were still higher compared with the specimen S3 irradiated with 100 keV-He^+^ to 2 × 10^20^ m^−2^, in which the damage dose and helium concentration were larger than those in S7. It suggested that higher damage dose and helium concentration were not the only reason for the larger amount of helium dissociated from S7. Based on the calculation of SRIM-2013, the ratio of concentrations of helium atoms to vacancies introduced by irradiation in S7 was higher than that in S2. It has been recognized that loop punching may occur for helium [34,35]. When n/m is larger than a criterion (n/m > 3, according to the literature [30]), the binding energy of a SIA with He_n_V_m_ clusters is smaller than that of a helium atom with He_n_V_m_ clusters. In this case, there could be the creation of vacancies and emission of SIAs. This is helpful for the growth of He_n_V_m_ clusters and helium bubbles and could contribute to the larger amounts of helium released from S7.

## 4. Conclusions

RAFM steels were irradiated with He^+^ with different energies to various fluences and at different temperatures. Specimens were examined with positron annihilation DB measurement and TDS to investigate the behavior of helium in RAFM steels. The mechanism was qualitatively elucidated based on rate equations. The findings are concluded as follows:

1. A threshold fluence of 2 × 10^20^ m^−2^ He^+^ was observed, above which the rate of formation and growth of helium bubbles dramatically increased, which was ascribed to the limitation of concentration of small He_n_V_m_ clusters when fluence is low.
2. Irradiation at the relatively higher temperature could suppress the formation and growth of He_n_V_m_ clusters with low binding energy and enhance that of helium bubbles and He_n_V_m_ clusters with high binding energy.
3. On increasing the irradiation temperature from 523 K to 723 K, the average n/m ratio of formed He_n_V_m_ clusters got closer to 1.3: it became larger and S parameters decreased at shallow depth; in contrast, the n/m ratio became smaller and S parameters increased at deep depth.
4. Irradiation of lower energy He^+^ enhanced the growth of He_n_V_m_ clusters and helium bubbles. It resulted from the larger ratio of helium atoms and vacancies introduced by irradiation and loop punching for He_n_V_m_ clusters with large n/m.

## Figures and Tables

**Table 1:** Chemical composition of the steel in the present study (wt. %).

Material	Si	P	Ti	V	Cr	Mn	W	C	S	N	Fe
**RAFM**	0.038	0.0074	<0.005	0.25	9.09	0.48	2.34	0.097	0.0019	0.035	Balance

**Table 2:** Irradiation conditions in present study.

Specimen	Ion Specie	Energy (keV)	Fluence (m^−2^)	Temperature (K)
S1	He^+^	100	2 × 10^19^	523
S2			1 × 10^20^	523
S3			2 × 10^20^	523
S4			5 × 10^20^	523
S5			2 × 10^21^	523
S6			1 × 10^20^	723
S7		18	1 × 10^20^	523
